# Stomatin-like protein 2 regulates survivin expression in non-small cell lung cancer cells through β-catenin signaling pathway

**DOI:** 10.1038/s41419-018-0461-9

**Published:** 2018-03-19

**Authors:** Cheng-Ta Yang, Jhy-Ming Li, Li-Fu Li, Yu-Shien Ko, Jeng-Ting Chen

**Affiliations:** 1 Department of Thoracic Medicine, Chang Gung Memorial Hospital, Taoyuan, Taiwan; 2 Department of Respiratory Therapy, College of Medicine, Chang Gung University, Taoyuan, Taiwan; 3 Division of Cardiology, Chang Gung Memorial Hospital, Taipei, Taiwan; 4 Department of Surgery, Chang Gung Memorial Hospital, Taoyuan, Taiwan

## Abstract

The overexpression of stomatin-like protein-2 (SLP-2) is commonly observed in non-small cell lung cancer (NSCLC) cells. In the present study, we transfected a number of NSCLC cells with an SLP-2 shRNA-expressing vector (AdSLP2i) and examined its possible effects on cell growth and apoptosis. We found that suppression of SLP-2 expression inhibited cell growth, and that the apoptosis induced by SLP-2 suppression was correlated with decreased survivin protein expression. Moreover, the reduced survivin expression was found to be associated with reduced β-catenin nuclear localization and appeared not to be modulated through the AKT signaling pathway. By using immunoprecipitation and proteomics to analyze protein–protein interactions in A549 cells with SLP-2 overexpression, we found that annexin A2 interacted with SLP-2 and β-catenin directly. Our data further suggested that the knockdown of SLP-2 gene affected the SLP-2/Annexin A2/β-catenin cascade formation, reduced the translocation of cytoplasmic β-catenin into nucleus, and downregulated downstream target genes. The results presented in this study, together with our previous findings, suggest that SLP-2 promotes NSCLC cell proliferation by enhancing survivin expression mediated via β-catenin pathway.

## Introduction

The stomatin gene superfamily consists of stomatin, stomatin-like protein-1 (*SLP-1*), *SLP-2*, and *SLP-3*^1–3^. Because of its homology to stomatin, SLP-2 was predicted to be cytoplasmically located and to link stomatin and/or other integral membrane proteins to the peripheral cytoskeleton^4^. In addition, SLP-2 was found to be upregulated by more than sixfold in esophageal squamous cell carcinoma (ESCC) tissues^5^ compared with their normal counterparts. Subsequently, the overexpression of *SLP-2* gene was also reported in human non-small cell lung cancer (NSCLC) cells, laryngeal carcinoma cells, and endometrial adenocarcinoma cells^6^. The same study also found that the transfection of SLP-2 antisense into ESCC cells suppressed cell growth and cell adhesion both in vitro and in vivo^6^. Furthermore, cell cycle analysis showed that the transfection of SLP-2 antisense led to S-phase arrest without apoptosis and the downregulation of fibronectin in ESCC cell lines^5,6^.

The frequent overexpression of SLP-2 in NSCLC cells suggests a role in carcinogenesis. Here we explored its signaling network and therapeutic potential in this malignancy. We constructed an adenoviral vector expressing small hairpin RNA (shRNA) against SLP-2 (AdSLP2i) to knockdown SLP-2 expression in NSCLC cells on a longer term basis. We found that the long-term suppression of SLP-2 expression in NSCLC cells resulted in cell apoptosis and, as such, we propose a possible regulatory mechanism explaining how SPL-2 regulates cell proliferation.

## Materials and methods

### Cell lines

The NSCLC cell lines A549 (ATCC CCL-185), H460 (ATCC HTB-177), H157 (ATCC CRL-5802), and H838 (ATCC CRL-5844), and the fetal lung fibroblasts cell line WI-38 (ATCC CCL-75) were purchased from American Type Culture Collection (ATCC, Manassas, Virginia, USA). Adult normal human lung fibroblasts cell line HLF were purchased from Clonetics (BioWhittaker, Inc., Walkersville, MD, USA).

### Adenoviral vectors

A 19nt SLP-2 targeting sequence, 5′-TCGACAATGTAACTCTGCAAA-3′, designed by SABioscience shRNA (catalog number KH07204G) was selected. A ring sequence of 6 base pairs (5′-ATCGAT-3′) existed between the sense and antisense strands. Using BLAST analysis, it was confirmed that the targeting sequence shared no homology with other coding sequences in the human genome. As described previously^7^, we used pUC-U6 plasmid and the pAdTrack vector to generate recombinant adenovirus expressing shRNA against SLP-2 (AdSLP2i). The recombinant adenovirus AdCtrl, which carries a green fluorescence protein (GFP) gene regulated by the cytomegalovirus (CMV) promoter was used as the control in these experiments^7^. The adenovirus vectors were amplified by using 293 cells and titered by using Adeno-X Rapid Titer Kit (BD Biosciences, San Jose, CA) in 293 cells.

### Development of stable A549 cell lines with high SLP-2 expression

To make the SLP-2 expression plasmid under the control of the CMV promoter (pCMVSLP2), a 1071 bp fragment of the human *SLP-2* gene was generated (nucleotides 64–1134, GenBank Accession Number NM_013442) by PCR reaction using A549 cDNA as the template. The oligonucleotide primers used were as follows: forward primer 5′-GAA ATG CTG GCG CGC GCG GCG CGG G-3′ and reverse primer 5′-CTA ACT CAT CTT GAC TCG ATC AAG C-3′. pcDNA3-SLP2 plasmid was constructed by subcloning the fragment of the entire SLP-2 encoding sequence from pJET1/blunt plasmid into the pcDNA3 between the *Xho*I and *Bam*HI sites. The A549 cells were subsequently transfected with pcDNA3-SLP2 using a liposome-mediated transfection technique with Lipofectamine 2000 reagent (Invitrogen, Grand Island, NY). Real-time PCR was used to screen the SLP-2 expression of the individual clones. The stable cell lines with high SLP-2 expression, namely A549SLP2 cells, were used for further experiments. In addition, A549EV was developed from A549 cells by transfection with empty vector pcDNA3 for the control studies.

### Western blotting

The cells (1 × 10^6^) were plated onto 10 cm dishes and incubated for 16 h at 37 °C before infection. The cells were subsequently mock-infected or infected with either AdCtrl or AdSLP2i, and incubated for 24~96 h. Total cell lysates were prepared with lysis buffer (M-PER Mammalian Protein Extraction Reagent; Thermo Fisher Scientific, Inc., Rockford, IL) and nuclear protein was then extracted using a nuclear extraction kit (AY2002; Panomics). The primary antibodies used were mouse anti-SLP-2 monoclonal antibody (60052-1-lg; Protein Tech Group, Inc.), rabbit anti-SLP-2 polyclonal antibody (sc-98709; Santa Cruz Biotechnology, Santa Cruz, CA), mouse anti-survivin monoclonal antibody (2802; Cell Signaling Technology), rabbit anti-poly-(ADP-ribose) polymerase (PARP) polyclonal antibody (9542; Cell Signaling Technology), mouse anti-active-β-catenin monoclonal antibody (anti-ABC; 05-665; Millipore), mouse anti-β-catenin monoclonal antibody (P35222; Millipore), rabbit anti-AKT polyclonal antibody (9272; Cell Signaling Technology), mouse anti-phospho-AKT (Ser473) monoclonal antibody (4051; Cell Signaling Technology), goat anti-γ tubulin polyclonal antibody (sc-7396; Santa Cruz Biotechnology), mouse anti-GAPDH monoclonal antibody (sc-47724; Santa Cruz Biotechnology), mouse anti-annexin A2 monoclonal antibody (sc-47696; Santa Cruz Biotechnology), and rabbit anti-annexin A2 polyclonal antibody (sc-9061; Santa Cruz Biotechnology), and horseradish peroxidase-conjugated goat anti-mouse, goat anti-rabbit, or donkey anti-goat antibody was used as the secondary antibody (Santa Cruz Biotechnology).

### Cell proliferation

The cells (1 × 10^5^/well) were plated in six-well culture plates and incubated for 16 h at 37 °C before infection. The cells were then mock-infected or infected with either AdCtrl or AdSLP2i at varying doses (multiplicity of infection [m.o.i.] of 10, 50, 100, and 200). On days 1, 2, 3, 4, 5, and 6 after infection, the cells were collected by trypsinization and suspended in phosphate-buffered saline (PBS). Viable cells were determined by direct microscopic counting using trypan blue exclusion. All counts were done on triplicate samples.

### Construction of survivin promoter plasmids

To construct luciferase expression plasmid under the control of survivin promoter (pSurvivin-Luc), a 977 bp fragment of the human *survivin* gene promoter was generated (nucleotides 1824–2800, GenBank Accession Number U75285) by PCR reaction from the A549 genomic DNA. The oligonucleotide primers used were as follows: forward primer 5′-ATA CGA GAT CTGG CCA TAG AAC CA-3′ and reverse primer 5′-ATG TAA AGC TTC CAC CTC TGC CA-3′. After the digestion and purification of restriction enzymes, the fragment was inserted into the luciferase vector pGL2-basic (Promega, Madison, WI) between the *Xho*I and *Hind*III sites. For an empty experimental control plasmid (pGL2-basic-CD), we used a promoter-less pGL2-basic vector in which the *luciferase* gene was replaced with the *survivin* promoter gene. These plasmids were confirmed by sequence analysis.

### Transient transfection and reporter assays

The luciferase reporter assay was used to normalize transfection efficiency. The cells were grown in 12-well plates with the suggested medium until 60–80% confluent. The cells were then transiently transfected with 1.6 μg of survivin promoter plasmid DNA or pGL2-basic plasmid DNA using 2 μl of Lipofectamine 2000 (Invitrogen) in 1 ml of Opti-Medium (Life Technology) per well for 4–6 h. Cell lysates were prepared 48 h after transfection. The light intensity was measured using a luminometer (MiniLumat LB 9506, Berthold) and all measurements were done with duplicate samples. The luciferase ratio was defined as the luciferase activity of the survivin promoter plasmids divided by the luciferase activity of pGL2-basic plasmid.

### RNA preparation and microarray assay

RNA was extracted from H838 cells that were infected with AdCtrl or AdSLP2i using Trizol according to the manufacturer’s protocol (Invitrogen). A microarray analysis was performed by Phalanx Biotech Group (Hsin-Chu, Taiwan). Briefly, total RNA (100 ng) was reverse transcribed to cDNA, amplified, labeled, and hybridized to the Human Genome U133 Plus 2.0 Array (Affymetrix, USA) according to the manufacturer’s instructions. After normalization of the expression values, the data from the AdSLP2i-transfected sample were compared with the AdCtrl-transfected sample. Data were considered significant when (1) the false discovery rate from the significance analysis of the microarray analysis for the comparison of knockdown and unknockdown expression values was < 0.05 and (2) the *p*-value of the comparison between the knockdown and unknockdown expression values by Student’s *t*-test was < 0.05. In order to focus on genes exhibiting a low degree of regulation, we restricted the majority of the analysis to genes with changes in expression levels of at least twofold.

### Assays for Rac1 activation

Activation of Rho family small GTPases was detected using an EZ-DetectRac1 Activation Kit (Pierce Biotechnology) and executed according to the manufacturer’s instructions.

### Immunofluorescence microscopy

The cells were infected with AdCtrl or AdSLP2i for 72 h on coverslips and grown to 50–80% confluence, fixed for 15 min at room temperature using 4% formaldehyde/PBS, and permeablized with 0.1% Triton X-100 in PBS. The cells were then blocked using 100% non-immune goat serum (50–062Z; Invitrogen) for 30 min and stained using anti-active-β-catenin monoclonal antibody (anti-ABC; 05–665; Merck). Primary antibodies were detected by rhodamine (TRITC)-conjugated donkey anti-mouse IgG antibodies (Santa Cruz Biotechnology). Immunofluorescence microscopy was performed using a confocal microscope with a 63 × NPL Fluotar objective.

### Immunoprecipitation of SLP-2 containing proteins

Immunoprecipitation was performed using agarose-conjugated rabbit polyclonal anti-SLP-2 antibody (Santa Cruz Biotechnology). The cell lysate was prepared from SLP-2 overexpressed A549 cells with a modified RIPA buffer (50 nM Tris-HCl, pH 7.4; 1% NP−40; 0.25% sodium deoxycholate; 150 nM NaCl; 1 mM EDTA; 1 mM phenylmethylsulfonyl fluorie; 1 μg/ml aprotinin, leupeptin, pepstatin, 1 mM Na_3_VO_4_; 1 mM NaF), and the protein concentration was adjusted to 1 μg/μl with PBS. Ten milligrams of cell lysate was added with 25 μl of anti-SLP-2 and incubated overnight. After microcentrifugation, the supernatant was discarded and the precipitated beads were washed three times with modified RIPA buffer, and the protein was dissociated from the beads with SDS-polyacrylamide gel electrophoresis sample buffer.

### Mass spectrometric analysis of gel-fractionated proteins

The protein bands of interest excised from silver-stained gels were destained by 1% potassium ferricyanide and 1.6% sodium thiosulfate, subjected to reduction and alkylation by 10 mM dithiothreitol/55 mM iodoacetamide in 25 mM NH_4_HCO_3_, and then in-gel digested with trypsin (20 μg/mL in 25 mM NH_4_HCO_3_) at 37 °C for 16 h. The reaction products were resolved with 0.1% Formic acid (FA) for liquid chromatography tandem mass spectrometry (LC-MS/MS) analysis. LC-MS/MS data acquisition was performed on a NanoLC U3000 (Dionex, Sunnyvale, CA) equipped with micrOTOFq (Bruker Daltonik GmbH, Bremen, Germany), with micrOTOFcontrol software. Samples were loaded into a trapping column (Agilent Zorbax 300SB-C18 was from Agilent Technologies (Santa Clara, CA) at 5 μl/min 100% elution buffer A (0.05% FA). After reducing the flow to 25 nl/min by using a splitter, peptides were separated by an analytical column (capillary RP18 column, Synergy hydro-RP, 2.5 μm, 0.15 × 100 mm, packed in-house) with a gradient from 5% to 80% elution buffer B (0.05% FA in 100% acetonitrile) in 75 min. Peptide fragment spectra were acquired from one MS scan, followed by four MS/MS scans of the more abundant parent ions. Each precursor was analyzed twice and then excluded in the following minute. LC-MS/MS data were calibrated and processed with DataAnalysis v.4.0 (Bruker Daltonics) for database search.

The emerging spectra identified via LC-MS/MS were used in searches of the uniprot_sprot201006 database (selected for *Homo sapiens*, 20 367 entries) assuming the digestion enzyme trypsin. The MASCOT search engine (http://www.matrixscience.com; v.2.2 Matrix Science) was used with two missing cleavage site with charge states from 1^+^ to 3^+^, MS mass tolerance was set to be 50 p.p.m. for LC-MS/MS and MS/MS tolerance was set to be 0.1 Da for both fix modification: Carbamidomethyl (C) and variable modifications: protein Acetyl (Protein N-term), Gln- > pyro-Glu (N-term Q), Oxidation (M). Protein identification was performed using Mowse scores (*p* < 0.05) and MudPIT algorithm of MASCOT search engine. The dat files were produced by Mascot Daemon and subjected to Scaffold (v.2.06.02; http://www.proteomesoftware.com) searching which assumed 99% protein probability, 95% peptide probability with two peptides minimum for protein and peptide identification.

### Statistical analyses

Results were expressed as means ± SE. Statistical comparisons were made with a two-sided t-test. A *p*-value < 0.05 was accepted as significant.

## Results

### AdSLP2i induced apoptosis in NSCLC cells

**Fig. 1:**
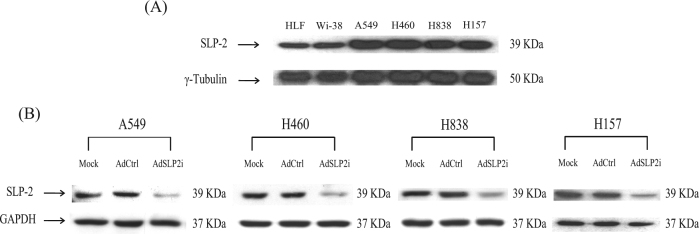
The expression of SLP-2 after AdSLP2i transfection in various cell lines. **a** SLP-2 expression levels in normal and NSCLC cells were analyzed by western blotting using antibodies against SLP-2 and γ-tubulin. The primary antibodies used are shown on the left of the panel. The cell lines are shown on the top of the panel. **b** Western blot assays for the expressions of SLP-2 protein in A549, H460, H838, and H157 NSCLC cells on day 3 after treatment with mock infection (Mock), or with either AdCtrl or AdSLP2i at m.o.i. of 100

The levels of SLP-2 expression in normal and NSCLC cell lines were evaluated by western blotting with anti-SLP-2-specific antibody. The expressions of SLP-2 in the NSCLC cell lines were much higher than those in normal HLF cell line (Fig. 1a). We then constructed an adenoviral vector, AdSLP2i, containing a U6 promoter to introduce ectopic SLP-2 shRNA expression to knockdown SLP-2 expression in NSCLC cells. We infected A549, H460, H838, and H157 NSCLC cells with AdSLP2i or AdCtrl at m.o.i. of 100 or conducted mock infection. After a 3-day period, the SLP-2 expression in the AdSLP2i-treated cells was markedly decreased compared with those in the AdCtrl- or mock-infected cells (Fig. 1b), indicating that AdSLP2i effectively inhibited SLP-2 expression in the NSCLC cell lines.

**Fig. 2:**
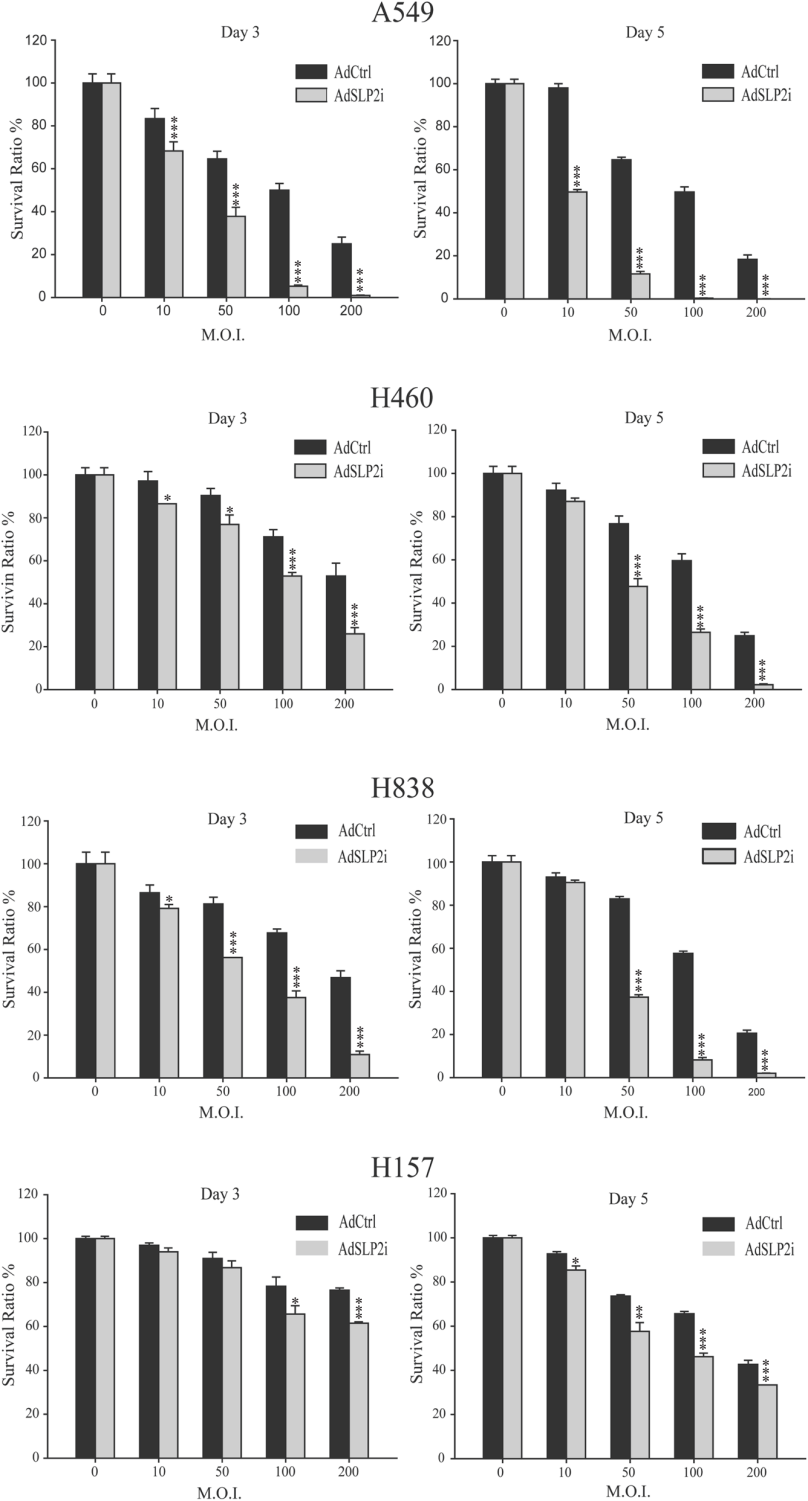
Cytotoxicity of AdSLP2i transfection on NSCLC cells. A549, H460, H838, and H157 cells were treated with varying doses of AdSLP2i or AdCtrl. Culture medium alone was used for mock infection. Triplet cultures were performed for each treatment and viable cells were counted on day 3 and day 5. The results are expressed as a percentage of the mock-infected cells (survival ratio). Points, mean; bars, SD

In order to find an optimal dose for the subsequent studies, we treated A549, H460, H838, and H157 cells with AdSLP2i or AdCtrl at 0, 10, 50, 100, or 200 m.o.i. As shown in Fig. 2, in the H460 and H383 cells there were no significant differences in survival ratio (i.e., the ratios of survival cell number in infected cells to the mock-infected cells) between AdSLP2i and AdCtrl transfection at m.o.i. of 10 examined on day 5 nor in H157 cells at m.o.i. of 10 and 50 on day 3. Of the other NSCLC cell lines examined, the numbers of viable cells in the AdSLP2i-infected group were significantly lower than those in the AdCtrl-infected groups at all m.o.i. tested (*p* < 0.05 in all comparisons). The cytotoxicity of AdSLP2i transfection was increased in a dose-dependent manner (Fig. 2); however, different NSCLC cell lines exhibited different sensitivities. At day 5, the ratios of viable cell numbers in A549, H460, H838, and H157 cells with AdCtrl treatment at m.o.i. of 100 to those subjected to mock infection were not < 50% (50.0 ± 1.36%, 59.5 ± 1.86%, 57.6 ± 0.63%, and 65.6 ± 0.00%, respectively) and the ratios of viable cell numbers in the AdSLP2i-infected cells treated at m.o.i. of 100 to those subjected to mock infection treatments were not > 50% (0.31 ± 0.05%, 26.4 ± 0.89%, 8.22 ± 0.63%, and 46.12 ± 0.91%, respectively). Therefore, we chose the m.o.i. of 100 to study the effect of AdSLP2i infection on the cell growth in the NSCLC cell lines.

The A549, H460, H838, and H157 cells were mock infected or infected with AdCtrl or AdSLP2i at m.o.i. of 100. The numbers of viable cells were counted from days 1 to 6 after transfection. The growth rates of the AdSLP2i-infected cells were substantively suppressed by SLP-2 gene silencing (Supplemental Fig. 1). From day 3 to day 6, the numbers of viable cells in the AdSLP2i-infected groups were significantly lower than those in the AdCtrl-infected groups in all the NSCLC cell lines examined (*p* < 0.05 in all comparisons). The results indicated that knockdown of SLP-2 expression by AdSLP2i treatment effectively inhibited NSCLC cell growth.

**Fig. 3:**
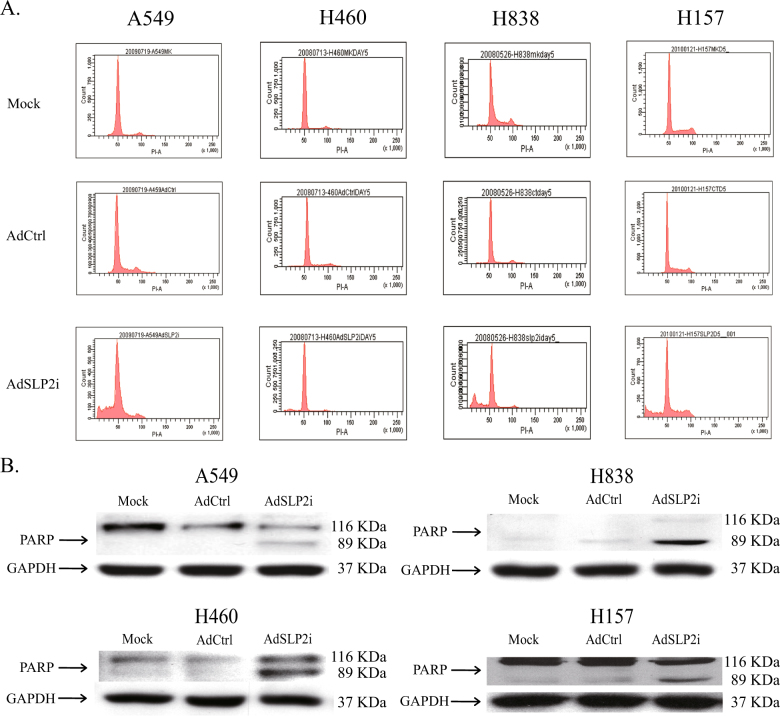
Cell apoptosis induced by knockdown of SLP-2 in NSCLC cells. **a** A549, H460, H838, and H157 cells were treated with mock infection or with either AdSLP2i or AdCtrl at m.o.i. of 100. On day 5 post infection, cell cycle analysis showed a marked increase in the subG1 population of the AdSLP2i-treated cells. **b** A549, H460, H838, and H157 cells were treated with either AdSLP2i or AdCtrl at m.o.i. of 100. On day 3 post infection, degradation of poly-(ADP-ribose) polymerase (PARP) was clearly detected by western blotting analysis in all four AdSLP2i-treated cell lines

To assess whether SLP-2 inhibition induces apoptosis, A549, H460, H838, and H157 cells were transfected with AdSLP2i or AdCtrl at m.o.i. of 100, or were subjected to mock transfection. On day 5 after the infection, cells were collected and cell cycle analysis was performed^7^. The results showed that there was a marked increase in the subG1 population of cells in the AdSLP2i-treated group (Fig. 3a). To confirm the presence of the apoptotic process, all the NSCLC cell lines were treated with either AdSLP2i or AdCtrl at m.o.i. of 100 and were examined for the presence of PARP cleavage by western blot analysis. From day 3 onwards, the degradation of PARP was clearly detected in the AdSLP2i-treated cells but not in the mock- nor AdCtrl-infected cells (Fig. 3b). The results indicated that SLP-2 inhibition by AdSLP2i transfection induced programmed cell death, as was evidenced by the increased subG1 cells and PARP protein cleavage.

### Microarray hybridization to identify gene(s) involved in SLP-2-related apoptosis

A cDNA microarray (Phalanx Biotech Group) was used to survey the probable factors involved in H838 cell apoptosis induced by SLP-2 inhibition. Marked downregulation of c-myc and survivin protein levels were observed on day 3 after transfection with AdSLP2i at m.o.i. of 100 compared with cells transfected with AdCtrl. Western blot analysis showed that in AdSLP2i-transfected human lung cancer cells, including A549, H460, H838, and H157 cells, the survivin protein expression levels were all suppressed (Supplemental Fig. 2A). To explore how SLP-2 regulates survivin expression, we cloned the *survivin* promoter gene and subcloned it into the upstream of the luciferase reporter gene in the pGL2-Basic vector (pGL2, Promega) to generate a new plasmid, pSurvivin-Luc. pSurvivin-Luc was then transfected into A549 cells; 4 h after that transfection, the cells were either mock transfected or transfected with AdCtrl or AdSLP2i at m.o.i. of 100. After incubation for 48 h, the cells were collected for luciferase assay. As shown in Supplemental Fig. 2B, the activity of survivin promoter in the AdCtrl-treated group was 4.33-folds higher than that in mock-infected group, and the activity of survivin promoter in AdSLP2i-treated group was 7.53% of that in AdCtrl-treated cells. These results indicated that AdSLP2i acted on the suppression of survivin gene transcription and thus decrease survivin protein levels.

### Investigation of the links between survivin and SLP-2

**Fig. 4:**
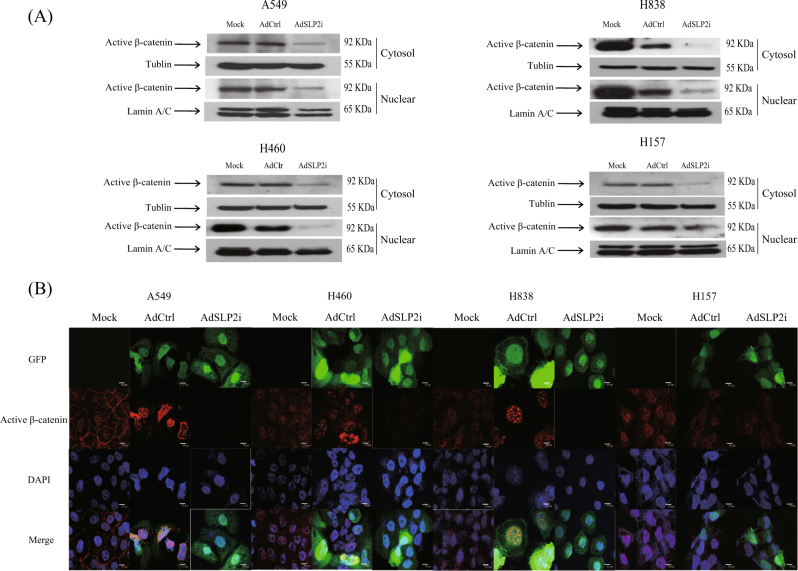
Western blotting and immunofluorescence analysis for the nuclear localization of β-catenin by SLP-2 inhibition in NSCLC cells. **a** Western blot analyses for the expressions of nuclear active β-catenin in A549, H460, H838, and H157 cells treated with either AdSLP2i or AdCtrl at m.o.i. of 100. **b** Immunofluorescence analyses for the nuclear localization of β-catenin in A549, H460, H838, and H157 cells treated with either AdSLP2i or AdCtrl at m.o.i. of 100. On day 3 post infection, the nuclear localization of β-catenin was clearly reduced in all four AdSLP2i-treated cell lines

Several signaling pathways have been suggested to be involved in the transcriptional control of survivin gene, including the Wnt and AKT signaling pathways^8^. The survivin gene is also an important target gene in the TCF-4/β-catenin signaling pathway^9^. In this regard, we found that in the cytosolic and nuclei of the AdSLP2i-transfected cells, β-catenin was decreased (Fig. 4a). Previous study has demonstrated that cyclin-dependent kinase 2-mediated β-catenin phosphorylation at residues Ser^23^, Ser^37^, Thr^41^, and Ser^45^ promotes rapid degradation of cytosolic β-catenin^10^. We further utilized the antibody recognizing active form of β-catenin with dephosphorylation on Ser^37^ or Thr^41^ (05-665, Anti-ABC, Merck) and immunofluorescence to confirm the change of β-catenin localization after AdSLP2i or AdCtrl-transfection (Fig. 4b). The results indicated that the nuclear content of the active form of β-catenin was consistently and obviously reduced in the AdSLP2i-transfected cells. These results suggested that survivin down-expression was probably regulated through the TCF-4/β-catenin signaling pathway.

**Fig. 5:**
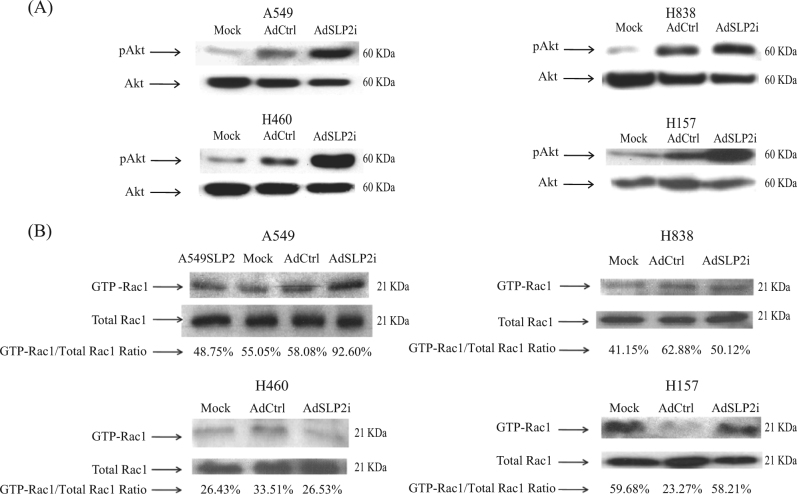
GTP-Rac1 and pAKT did not participate in TCF/β-catenin signaling pathway after SLP-2 inhibition in NSCLC cells. **a** Western blot analyses for the expressions of pAkt in A549, H460, H838, and H157 cells treated with either AdSLP2i or AdCtrl at m.o.i. of 100. **b** Downregulation of SLP-2 increased the GTP-Rac1 proteins in A549 and H157 cells, but decreased the GTP-Rac1 proteins in H838 and H460 cells as determined by pull-down assay. Total cell lysates were subjected to the pull-down assay for GTP-Rac1 activities. Total cell lysates were analyzed for Rac1 expression as a loading control. The ratio of GTP-Rac1/total Rac1 in cells was analyzed by densitometry of the blot

We further examined AKT expression in NSCLC cell lines after either mock transfection or transfection with AdSLP2i or AdCtrl at m.o.i. of 100. Three days after AdSLP2i transfection, phospho-AKT (pAKT) was found to be up-regulated in the AdSLP2i-transfected cells compared with the cells in the other two experimental groups (Fig. 5a).

To confirm that survivin, pAKT, and β-catenin were regulated by SLP-2 inhibition, we generated a SLP-2 overexpression cell line, A549SLP2, to study this issue. The A549SLP2 cell line was established by the stable transfection of CMV promoter-controlled SLP-2 expression vector. The expression of SLP-2 in the A549SLP2 cells was markedly higher than that in A549EV cells. We found that the nuclear β-catenin and survivin were up-regulated and that pAKT was downregulated in the A549SLP2 cells compared to the A549EV cells (Supplemental Fig. 3A-D). These results further established that the nuclear translocation of β-catenin affected survivin expression and that it was regulated by SLP-2 expression status.

A previous report indicated *Rho-GTPases* regulate cytoskeleton by becoming inactive when GDP-bound or by becoming active when GTP-bound^11^. Another study found that Rac1 is a member of the Rho family that can controll the nuclear localization of β-catenin via the Wnt signal pathway^12^. In this study, we sought to investigate whether the *Rac1-GTPase* could control the nuclear localization of β-catenin to mediate survivin expression after AdSLP2i treatment. Our data indicated that *Rac1-GTPase* was active in A549 and H157 cells but inactive in H460 and H838 cells after cells of each type were treated with AdSLP2i (Fig. 5b). These results indicated that *Rac1-GTPase* did not participate the in TCF/β-catenin signaling pathway.

### Immunoprecipitation and proteomics to analyze protein–protein interactions in cells with SLP-2 overexpression

**Fig. 6:**
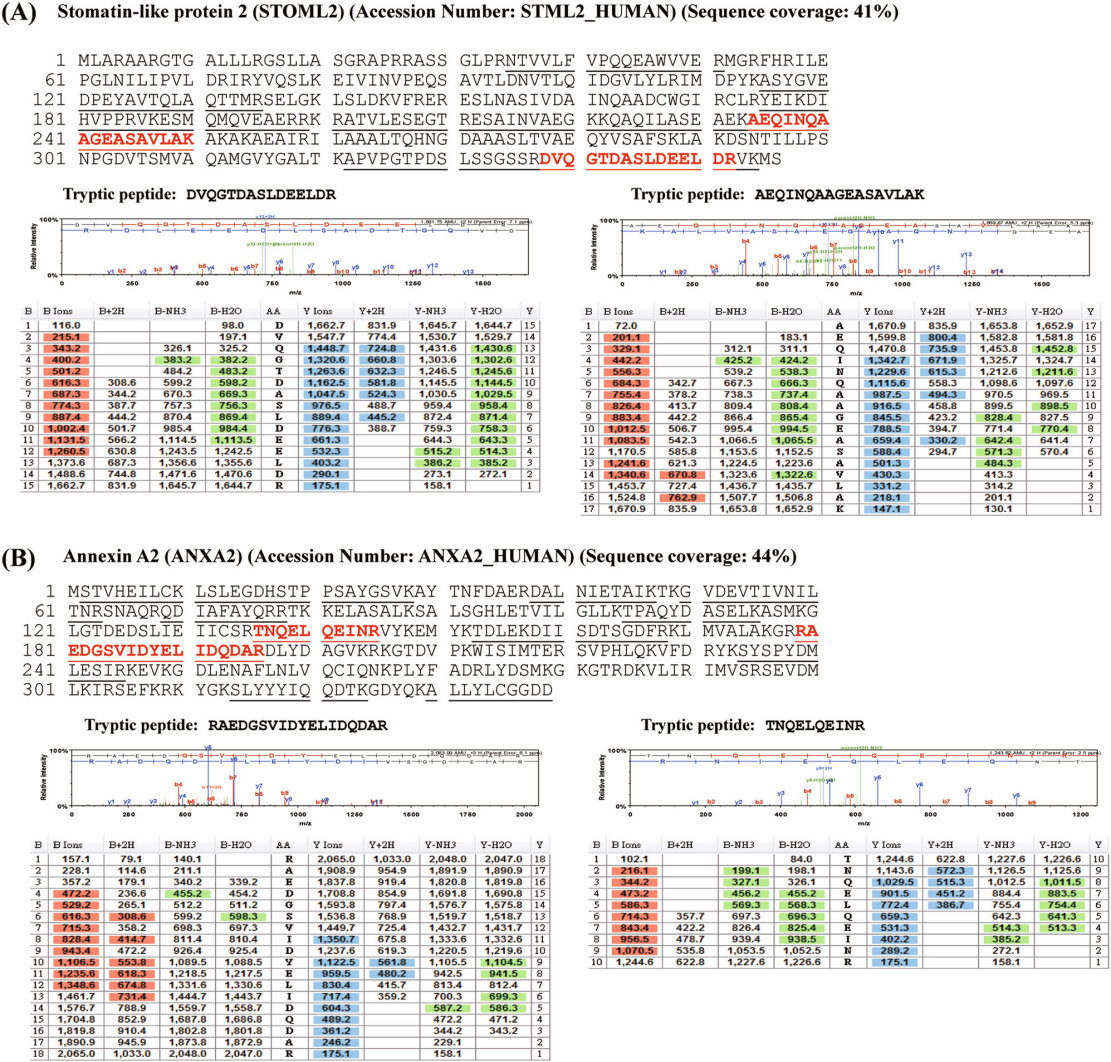
Representative MS/MS spectra from micrOTOFq used to confirm the identification of STOML2 interactome. Tryptic peptides of protein bands in the SDS-gel were analyzed by LC-MS/MS using micrOTOFq. The amino acid sequences of peptides identified by MS/MS analysis and matched to the amino acid sequences of proteins are underlined. The representative MS/MS spectra of two selected peptides (indicated in blood red) for each protein are also denoted below the amino acid sequences. The assignments of the fragmented ions observed to specific amino acid residues were performed using Scadffold 2 search engine, and the search results are shown below the MS/MS spectra

Our data showed that the knockdown of SLP-2 gene inhibited survivin expression via the TCF-4/β-catenin signaling pathway, but not via PI3K/Akt-Rac1 signaling. By then using LC-MS/MS and the database, we detected the interaction between SLP-2 and annexin A2 in A549SLP2 cells (Fig. 6a, b). We further used immunoprecipitation and western blotting to confirm if there were interactions among SLP-2, annexin A2, and β-catenin. Our results indicated that annexin A2 interacted with SLP-2 and β-catenin directly (Supplemental Fig. 4A). In addition, we found that the expression of annexin A2 was not changed by the knockdown or overexpression of SLP-2 gene (Supplemental Fig. 4B).

## Discussion

The *SLP-2* is a novel oncogene that is over-expressed in human ESCC cells, NSCLC cells, laryngeal carcinoma cells, and endometrial adenocarcinoma cells^6^. In the present study, we first analyzed the expression of SLP-2 in normal and NSCLC cells using western blotting and, as expected, the expression of SLP-2 in the NSCLC cells was much higher than that in the normal cells. We then designed an adenovirus vector, designated as AdSLP2i, harboring SLP-2 shRNA under the control of a U6 promoter in order to silence the expression of SLP-2 in NSCLC cells. The cell survival was found to be greatly reduced in H460, A549, and H838 cells transfected with AdSLP2i at various m.o.i. on day 3 and day 5 compared with the cells in the AdCtrl group (Fig. 2). However, cell death induced by AdSLP2i transfection was not apparent in H157 cells nor was cell growth suppression induced by AdSLP2i transfection apparent in H460 and H157 cells. These data might suggest that the inhibition of SLP-2 expression reduced cell proliferation, and that this suppressive effect varied in different cell types. Moreover, the suppression of SLP-2 expression resulted in PARP cleavage and cell apoptosis, and both the microarray analysis and western blotting showed a correlation between the decrease of survivin expression and cell apoptosis. The results observed in this study, together with our previous report^7^, suggest that the following regulatory circuit is involved in the regulation of NSCLC cell growth: SLP-2 repression leads to reduced survivin activity, which in turn results in PARP cleavage and then culminates in cell apoptosis.

The expression of survivin have been reported to be regulated by several signal pathways including TCF-4/β-catenin and PI3K/AKT-Rac1^8,9,11,12^. The TCF-4/β-catenin pathway has an important role in the Wnt signaling pathway. The activation of Wnt signaling enhances the translocation of β-catenin into cell nuclei, where it binds to transcription factors of the TCF/LEF family. The β-catenin-TCF/LEF complex then induces transcription of downstream target genes in cancer, such as c-Myc, cyclin D1, and Tcf-1^13^, and subsequently lead to the transactivation of survivin promoter^14^.

The PI3K/AKT signaling pathway has been shown to regulate the cell survival, cell cycle progression, and cellular growth of many types of cancer cells. The activation of PI3K/AKT was found to promote β-catenin phosphorylation^15^, whereas the transfection of CA-AKT DNA into cells has been shown to increase the expression of both pAKT and survivin^16^. The results of the present study, however, showed that pAKT was upregulated but that nuclear β-catenin was downregulated after AdSLP2i transfection (Fig. 4a, b). In contrast, the increase of SLP-2 protein reduced pAKT expression but increased nuclear β-catenin expression (Supplemental Fig. 3A-D). These results strongly suggest that β-catenin directly regulates survivin expression through the TCF-4/β-catenin signaling pathway rather than through the PI3k/AKT signaling pathway.

Wu et al.^12^ reported that Rac1-GTPase activation enhances the nuclear localization of β-catenin in the Wnt signaling pathway and that the knockdown of the expression of Rho family small GTPases stimulates AKT1 upregulation, leading to enhanced cell migration and invasion in human bronchial cells^17^. Our data showed that the knockdown of SLP-2 upregulated pAKT expression and downregulated nuclear β-catenin. In the present study, we assessed Rac1-GTPase activity by western blotting after AdSLP2i transfection. We found that Rac1-GTPase was upregulated in A549 and H157 cells but was downregulated in H460 and H838 cells (Fig. 5a–d). These results indicated that β-catenin translocation into nuclei was probably not regulated by Rac1-GTPase activation. Further analysis by immune-precipitation and proteomics showed direct interactions among SLP-2, β-catenin, and annexin A2, suggesting a role of annexin A2 in this regulatory cascade.

Overexpression of annexin A2 has been found in many cancers, such as NSCLC cancer^18^, Lewis lung cancer^19^, gliomas^20^, gastric cancer^21^, brain tumors^22,23^, acute promyelocytic leukemia^24^, colorectal carcinoma^25^, and pancreatic cancer^26^. Relatedly, the knockdown of annexin A2 gene was reported to inhibit cell division and proliferation by blocking DNA synthesis^27,28^, reducing the levels of p11 and c-Myc, and causing cell cycle arrest^29^. Moreover, the downregulation of annexin A2 has been shown to reduce the activation of β-catenin and the expression of cyclin D1^30,31^. Our data suggested that SLP-2/Annexin A2/β-catenin cascade formation might prevent the phosphorylation and subsequent degradation of β-catenin, so the inhibition of SLP-2 expression might reduce the accumulation and translocation of cytoplasmic β-catenin into nuclei, and thus downregulated downstream target genes such as survivin and cyclin D1.

In summary, the molecular mechanisms of SLP-2 regulation in cancer remain unclear. Our results suggested that SLP-2 promotes NSCLC cell proliferation by enhancing survivin expression mediated through the TCF4/β-catenin pathway (Supplemental Fig. 5). We also suggest that the β-catenin pathway, but not the PI3K/AKT pathway, is modulated by SLP-2 in NSCLC cells.

## Electronic supplementary material

Supplemental Figure 1 Inhibition of NSCLC cell growth after AdSLP2i transfection(PDF 1075 kb)

Supplemental Figure 2 The expression of survivin after AdSLP2i transfection of NSCLC cells(PDF 551 kb)

Supplemental Figure 3 Expression of pAkt, nuclear active β-catenin, and survivin in A549SLP2 cell by SLP-2 overexpression(PDF 594 kb)

Supplemental Figure 4 Correlations of SLP-2, annexin A2, and nuclear active β-catenin protein-protein interactions in A549SLP2 cells(PDF 1544 kb)

Supplemental Figure 5 The proposed mechanisms of SLP-2 regulation in NSCLC cells(PDF 3835 kb)

Supplementary Figure Legends(DOC 45 kb)

Gene expression data on microarray after H838-infected with AdCtrl and AdSLP2i for 3 days(XLS 18248 kb)
