# Supplementary figures and images for: Stomatin-like protein 2 regulates survivin expression in non-small cell lung cancer cells through β-catenin signaling pathway
Source: Cell Death Dis. 2018 Mar 19;9(4):425. doi: 10.1038/s41419-018-0461-9 (PMC5859036; doi:10.1038/s41419-018-0461-9)

A549

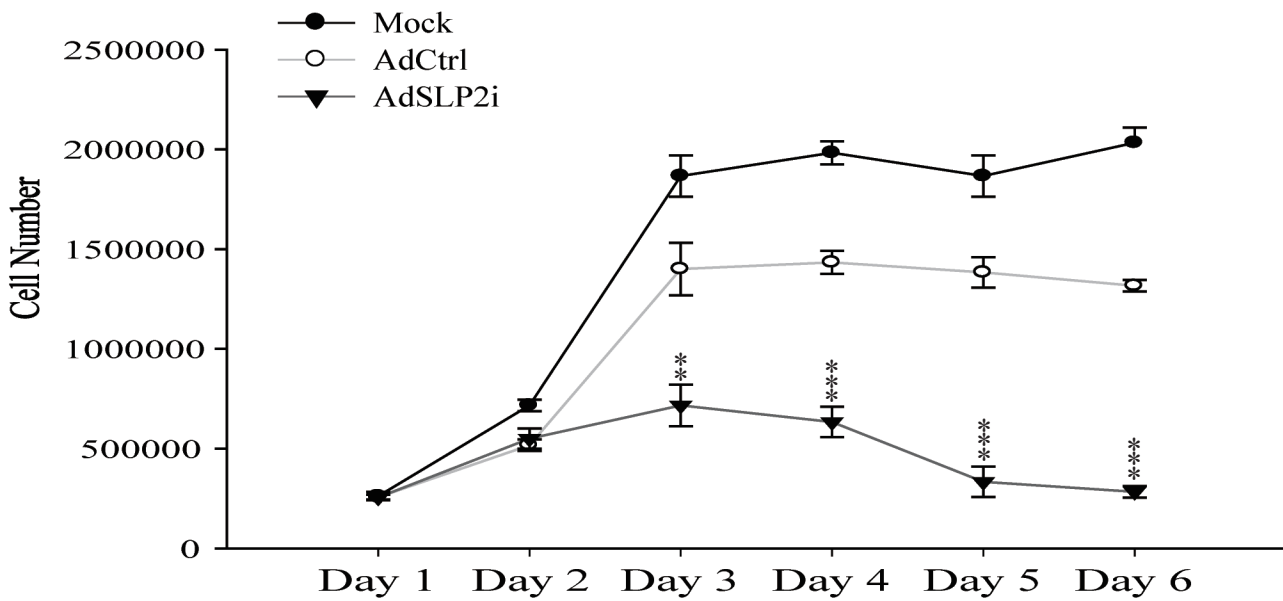

H838

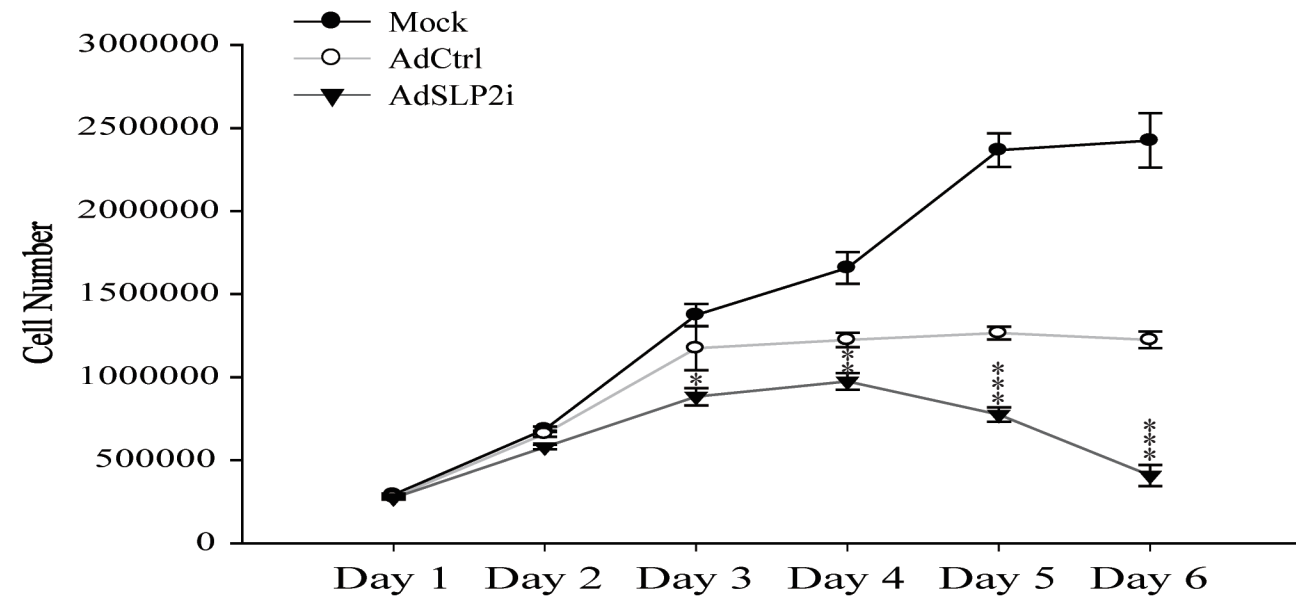

H460

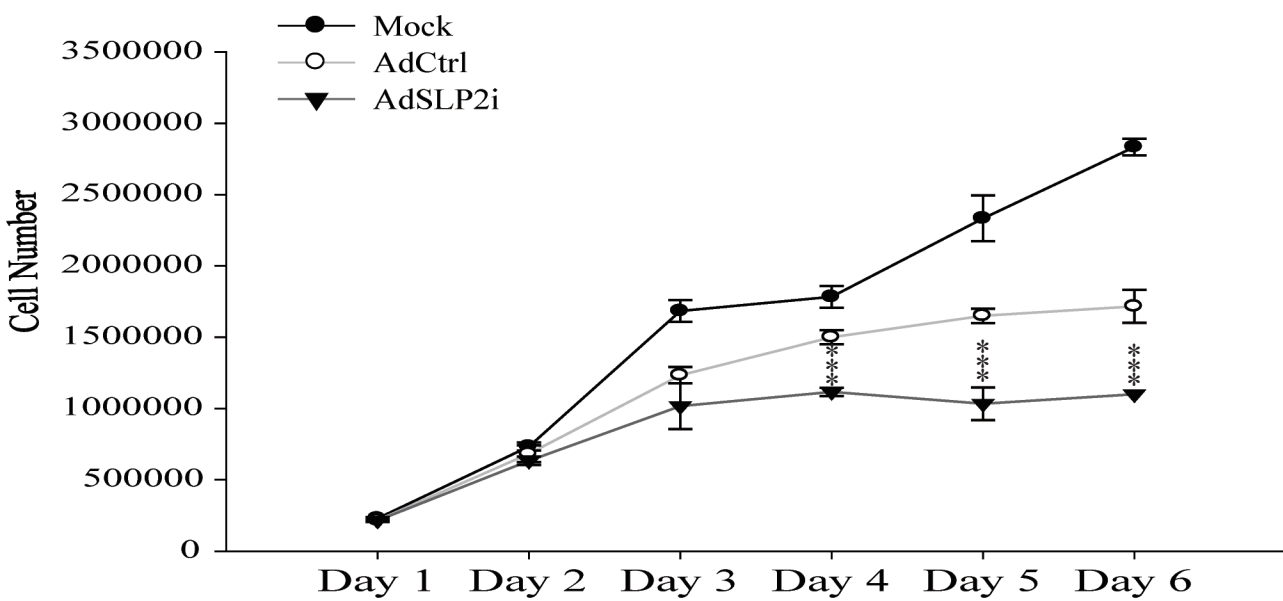

H157

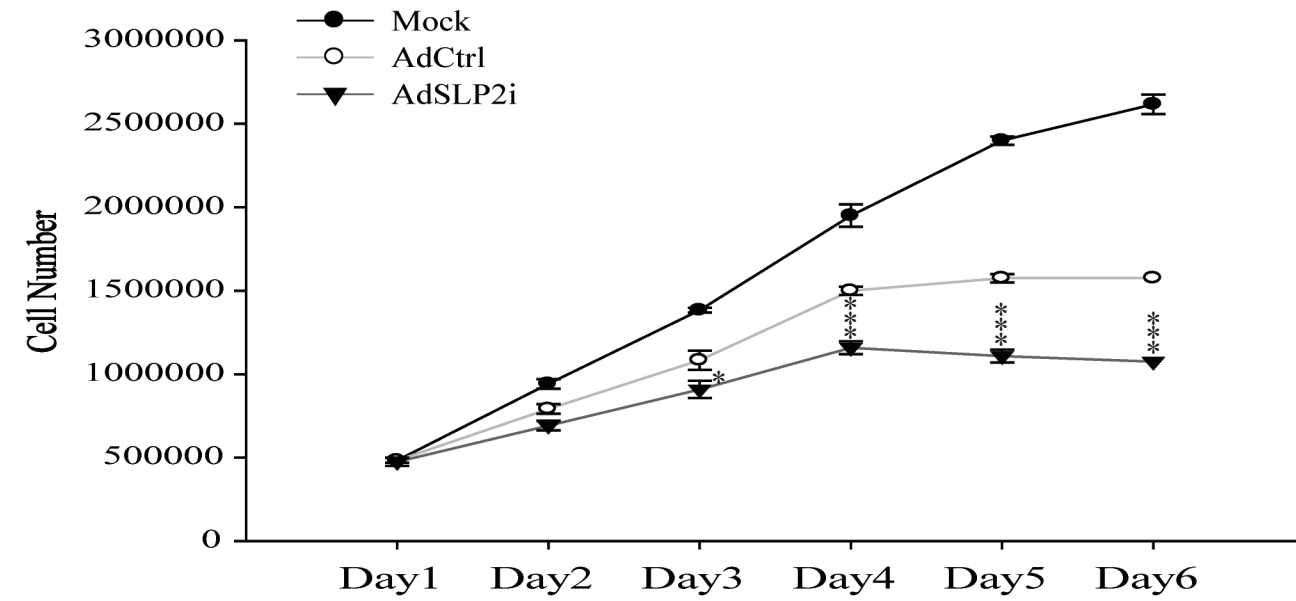

Supplement: Supplementary file 1 — Supplemental Figure 1 Inhibition of NSCLC cell growth after AdSLP2i transfection(PDF 1075 kb) [file 41419_2018_461_MOESM1_ESM.pdf]

A.

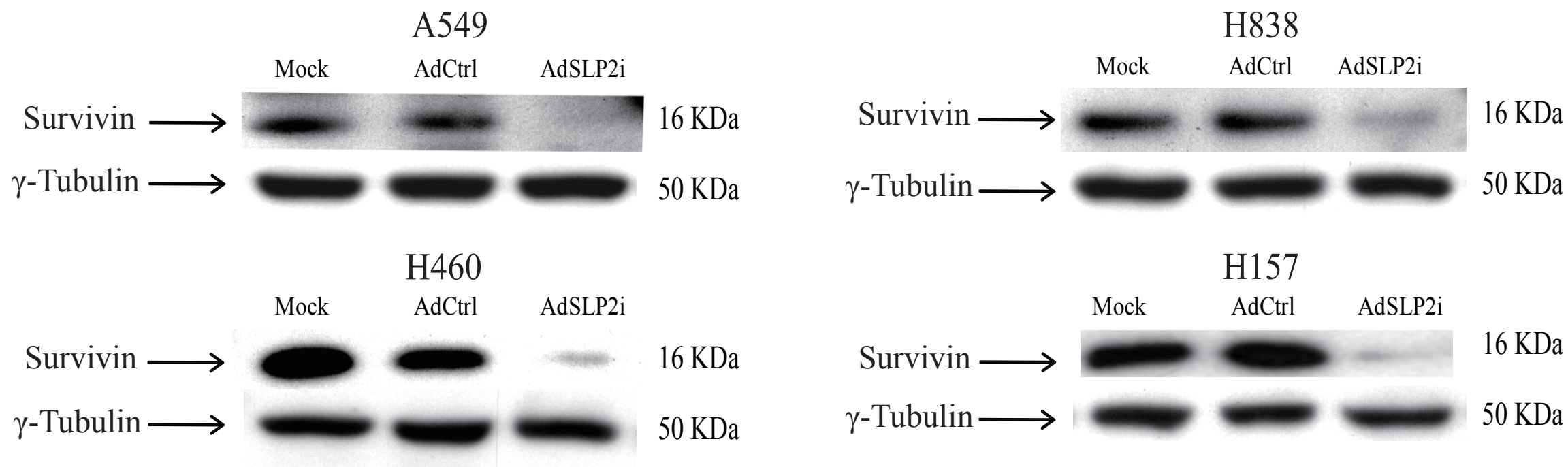

B.

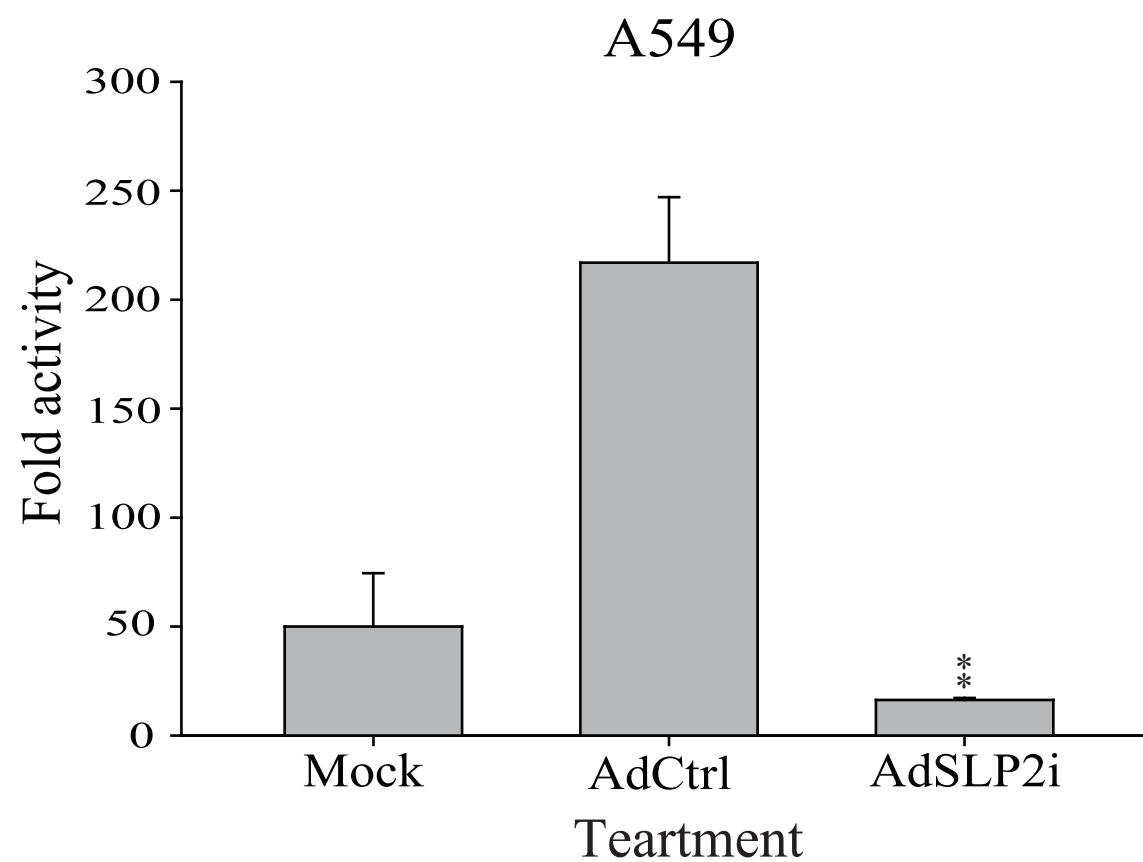

Supplement: Supplementary file 2 — Supplemental Figure 2 The expression of survivin after AdSLP2i transfection of NSCLC cells(PDF 551 kb) [file 41419_2018_461_MOESM2_ESM.pdf]

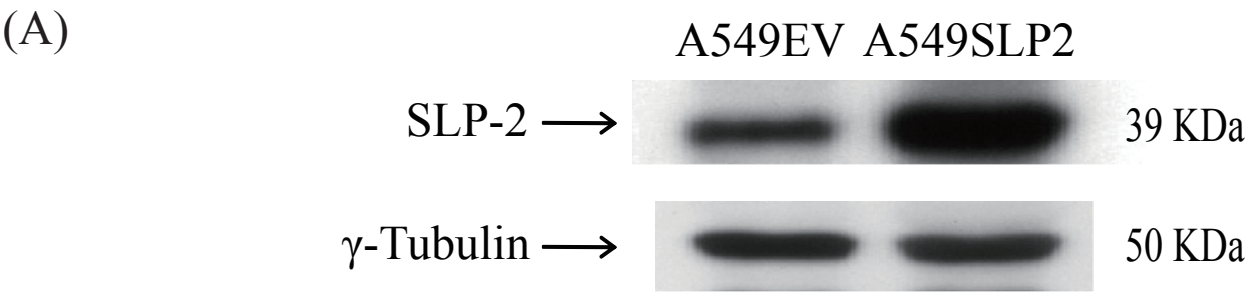

$A549SLP2/A549EV=2.74$

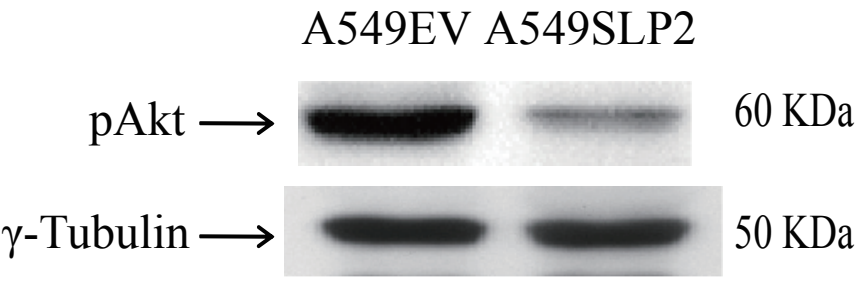

$A549SLP2/A549EV=0.34$

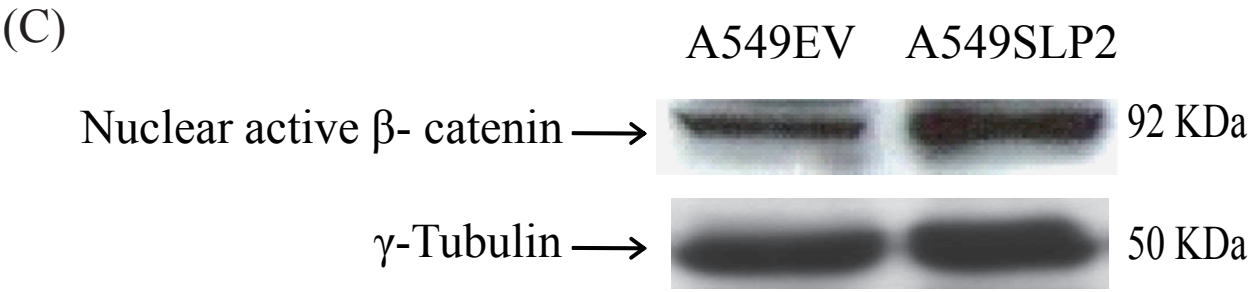

$A549SLP2/A549EV=1.54$

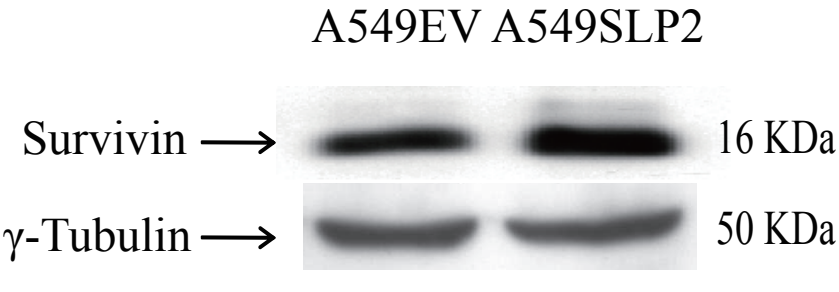

$A549SLP2/A549EV=1.37$

Supplement: Supplementary file 3 — Supplemental Figure 3 Expression of pAkt, nuclear active β-catenin, and survivin in A549SLP2 cell by SLP-2 overexpression(PDF 594 kb) [file 41419_2018_461_MOESM3_ESM.pdf]

(A)

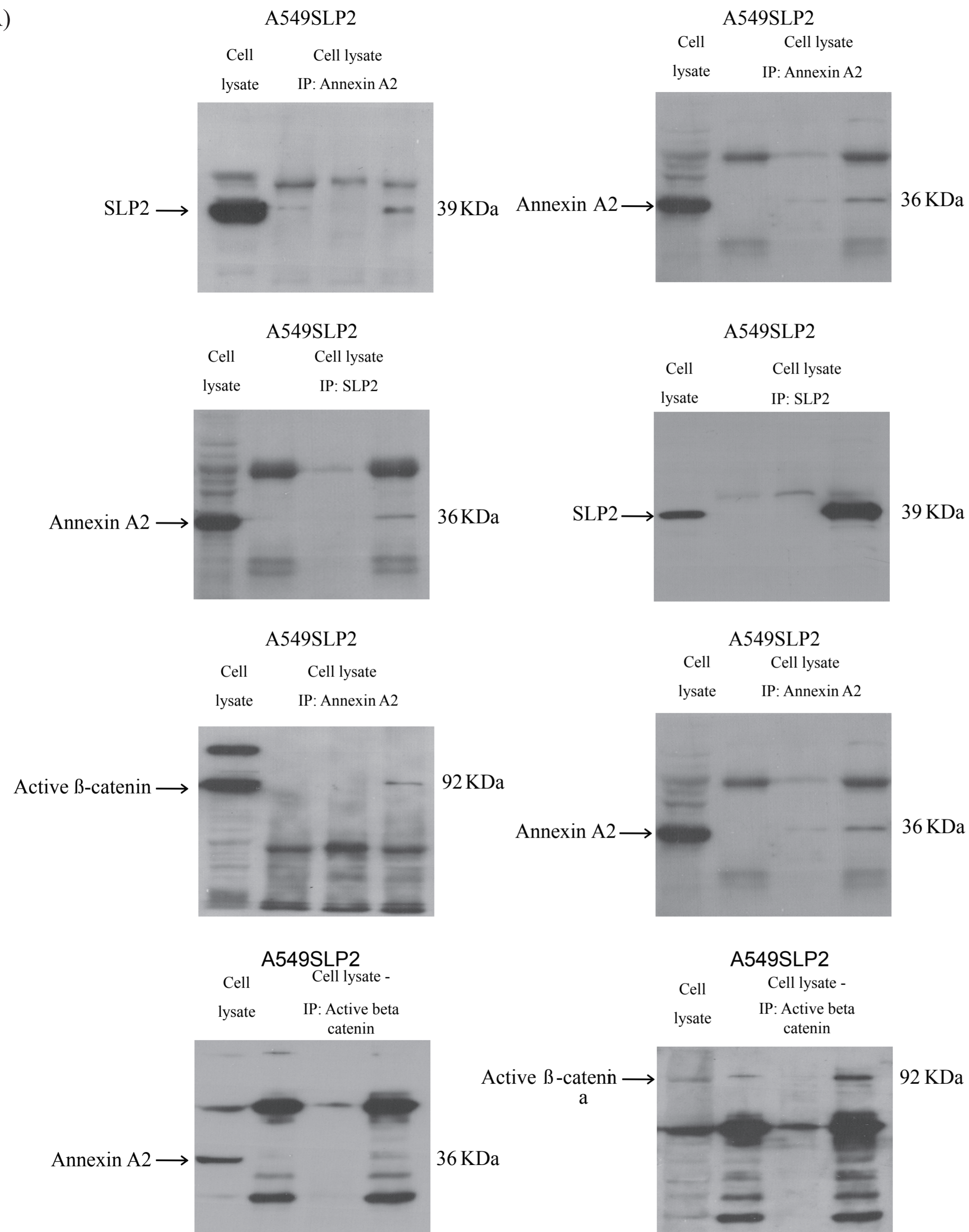

(B)

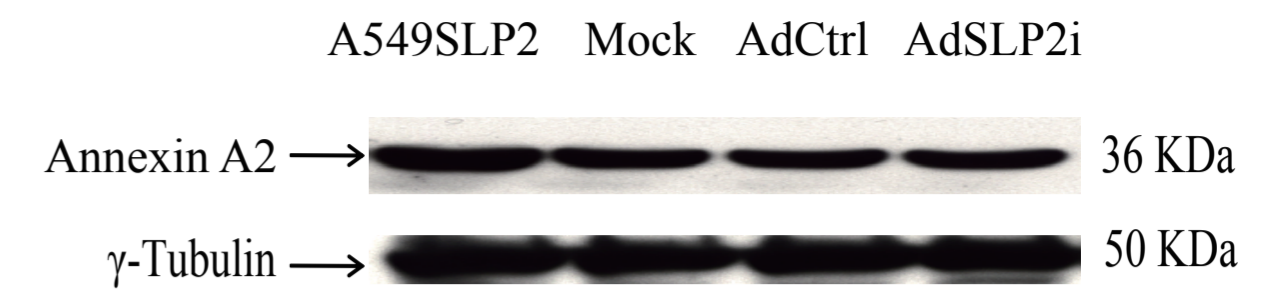

Supplement: Supplementary file 4 — Supplemental Figure 4 Correlations of SLP-2, annexin A2, and nuclear active β-catenin protein-protein interactions in A549SLP2 cells(PDF 1544 kb) [file 41419_2018_461_MOESM4_ESM.pdf]

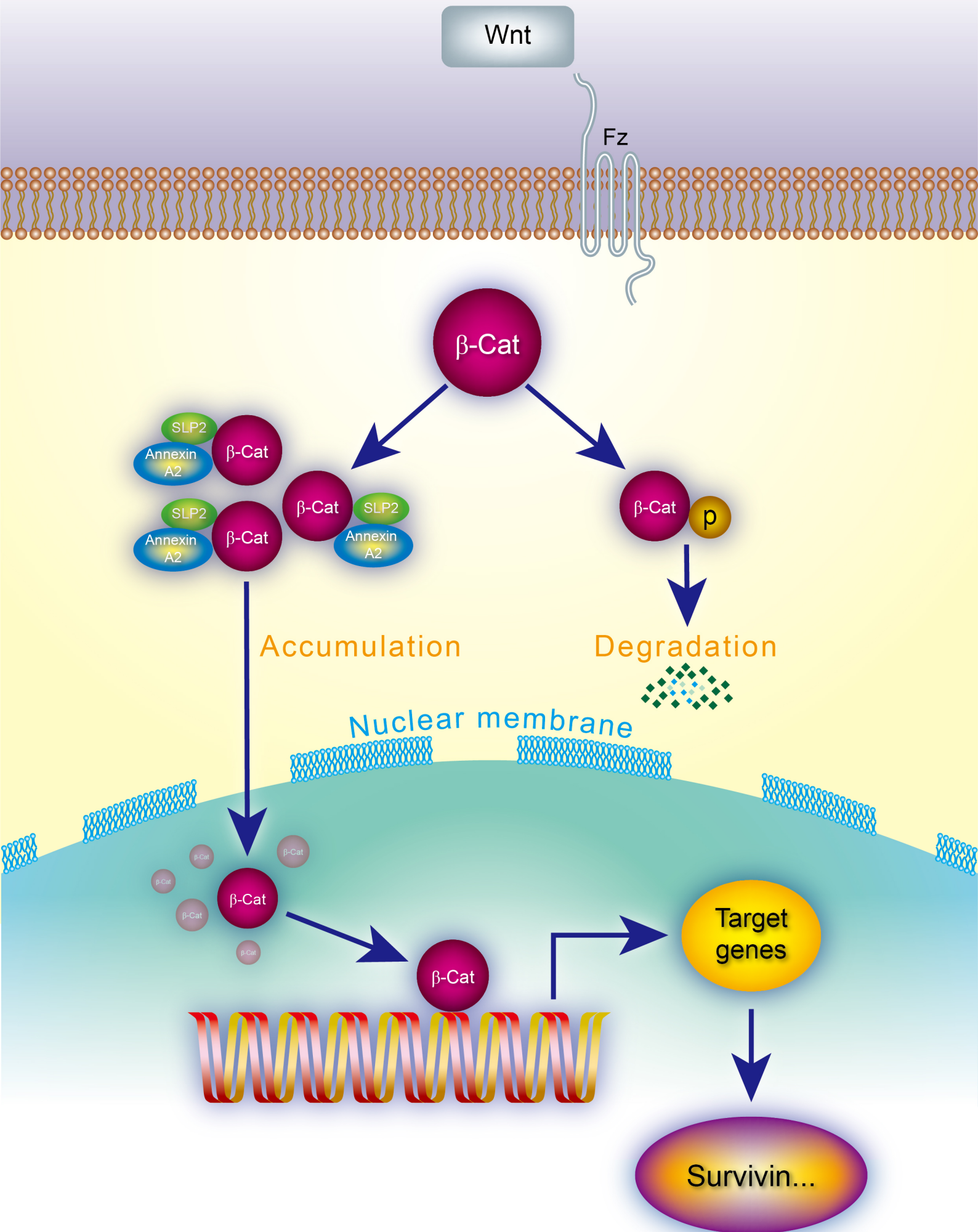

Supplement: Supplementary file 5 — Supplemental Figure 5 The proposed mechanisms of SLP-2 regulation in NSCLC cells(PDF 3835 kb) [file 41419_2018_461_MOESM5_ESM.pdf]
