# Supplementary material for: Stomatin-like protein 2 regulates survivin expression in non-small cell lung cancer cells through β-catenin signaling pathway
Source: Cell Death Dis. 2018 Mar 19;9(4):425. doi: 10.1038/s41419-018-0461-9 (PMC5859036; doi:10.1038/s41419-018-0461-9)
Supplement: Supplementary file 6 — Supplementary Figure Legends(DOC 45 kb) [file 41419_2018_461_MOESM6_ESM.doc]

**Supplementary Figure Legends**

**Supplemental Figure 1 Inhibition of NSCLC cell growth after AdSLP2i transfection.** A549, H460, H838, and H157 cells were treated with AdSLP2i or AdCtrl at m.o.i. of 100. Culture medium alone was used for mock infection. Triplet cultures for each treatment were counted daily for viable cells on post-infection days 1-5. The results are expressed as a percent of the mock-infected cells (survival ratio). Points, mean; bars, s.d.

**Supplemental Figure 2 The expression of survivin after AdSLP2i transfection of NSCLC cells.** **(A)** Western blot assays for the expression of survivin in A549, H460, H838, and H157 NSCLC cells on day 3 after treatment with mock infection (Mock), or with either AdCtrl or AdSLP2i at m.o.i. of 100. **(B)** Report assays for the activity of survivin promoter, the survivin promoter DNA was transiently transfected into A549 cell. After transient transfection for 24 hours, cells were treated with mock infection (Mock), or with either AdCtrl or AdSLP2i at m.o.i. of 100.

**Supplemental Figure 3 Expression of pAkt, nuclear active β-catenin, and survivin in A549SLP2 cell by SLP-2 overexpression.** Western blot assays for the expressions of SLP-2, pAkt, nuclear active β-catenin, and survivin in A549SLP2 cells. **(A)** The SLP-2 was overexpressed in the A549SLP2 cells relative to the A549EV cells. Furthermore, the (**B**) pAkt was downregulated, while **(C)** nuclear active β-catenin and **(D)** survivin were upregulated in the A549SLP2 cells relative to the A549EV cells.

**Supplemental Figure 4 Correlations of SLP-2, annexin A2, and nuclear active β-catenin protein-protein interactions in A549SLP2 cells. (A)** Immuno-precipitation (IP) assays for the SLP-2, annexin A2, and nuclear active β-catenin protein-protein interactions in A549SLP2 cells. **(B)** Expression of annexin A2 in A549 cells by SLP-2 inhibition. Western blot assays for the expressions of annexin A2 protein in A549 cells on day 3 after treatment with mock infection (Mock), or with either AdCtrl or AdSLP2i at m.o.i. of 100.

**Supplemental Figure 5 The proposed mechanisms of SLP-2 regulation in NSCLC cells.** SLP-2/Annexin A2/β-catenin cascade formation might prevent cytosolic β-catenin from phosphorylation and degradation, and then promote NSCLC cell proliferation by enhancing target genes, including survivin, expression. Downregulation of SLP-2 expression might thus inhibit cell growth and lead to apoptosis.
